# Perceptual Strategies of Pigeons to Detect a Rotational Centre—A Hint for Star Compass Learning?

**DOI:** 10.1371/journal.pone.0119919

**Published:** 2015-03-25

**Authors:** Bianca Alert, Andreas Michalik, Sascha Helduser, Henrik Mouritsen, Onur Güntürkün

**Affiliations:** 1 Institut für Biologie und Umweltwissenschaften, Carl von Ossietzky Universität Oldenburg, D-26111, Oldenburg, Germany; 2 Research Centre Neurosensory Science, University of Oldenburg, D-26111, Oldenburg, Germany; 3 Department of Psychology, Institute of Cognitive Neuroscience, Biopsychology, Ruhr-University Bochum, D-44780, Bochum, Germany; Lund University, SWEDEN

## Abstract

Birds can rely on a variety of cues for orientation during migration and homing. Celestial rotation provides the key information for the development of a functioning star and/or sun compass. This celestial compass seems to be the primary reference for calibrating the other orientation systems including the magnetic compass. Thus, detection of the celestial rotational axis is crucial for bird orientation. Here, we use operant conditioning to demonstrate that homing pigeons can principally learn to detect a rotational centre in a rotating dot pattern and we examine their behavioural response strategies in a series of experiments. Initially, most pigeons applied a strategy based on local stimulus information such as movement characteristics of single dots. One pigeon seemed to immediately ignore eccentric stationary dots. After special training, all pigeons could shift their attention to more global cues, which implies that pigeons can learn the concept of a rotational axis. In our experiments, the ability to precisely locate the rotational centre was strongly dependent on the rotational velocity of the dot pattern and it crashed at velocities that were still much faster than natural celestial rotation. We therefore suggest that the axis of the very slow, natural, celestial rotation could be perceived by birds through the movement itself, but that a time-delayed pattern comparison should also be considered as a very likely alternative strategy.

## Introduction

Birds can use a variety of orientation cues to find their way between their breeding and wintering grounds and for homing from an unfamiliar location. The Earth’s magnetic field [1–5] and/or celestial cues [6–12] provide important cues for compass orientation [2,13–15]. Traditionally, birds are considered to have two compasses based on celestial cues: a sun azimuth compass [8,16,17] and a star compass [7,9,10,18,19]. In both of these compasses, the axis of celestial rotation seems to play a crucial role, and it is known that hand-raised birds can calibrate their magnetic compass by observing either the day-time sky on which the sun and the polarised light patterns rotate [11,20] or the night-time sky on which the stars rotate [10,21]. Therefore, it cannot be excluded that the sun and the star compasses are parts of a single celestial compass system in birds.

In fact, birds seem to possess the inherent information to look for rotating light dots in the sky during a sensitive period and to interpret the centre of rotation as North [10,18,19]. Once the centre of rotation has been determined, birds can show appropriate migratory orientation by means of their star compass even under a stationary starry sky [10,12,19,22] and they can also calibrate their magnetic compass accordingly [21,23–26]. Birds seem to have no preconceived ideas about how the star patterns should look like because even an arbitrary star pattern consisting of just 16 diodes seems to be accepted as the stars by naïve birds [19,21,22,26].

Hence, the ability to detect a centre of rotation of a group of light dots seems to be a crucial ability for many birds in order to establish a functional celestial compass. However, so far, very little is known about the abilities of birds to detect rotational centres. In fact, it has never been shown directly that birds can learn the concept of a rotational centre, and if they can learn it, which strategy they use. The perceptual strategies employed during this learning process are possibly inherent to bird vision. We therefore decided to use homing pigeons (*Columba livia*) as experimental animals because of their ability to quickly learn diverse visual operant conditioning tasks, including those relating to moving stimuli [27–30].

From a psychophysical perspective, pigeons could detect the rotational axis of a rotating dot pattern by applying one of two fundamentally different strategies. They could either use local features of the stimulus, such as a single slowly or non-moving dot, or the global stimulus configuration to deduce its rotational centre. The processing of hierarchical stimuli which contain local as well as global information is one of the key issues in visual cognition (see [31] for summary). In humans, the global stimulus configuration usually receives a higher priority [32,33] (see [34] for review) whereas pigeons commonly show visual precedence for local stimulus cues [29,31,35–37] despite their general ability to use global cues as well [30,38]. Thus, pigeons are generally sensitive to both levels of stimulus organisation and they can also be trained to shift their attention from local to global cues [39,40].

In our rotating dot patterns, the local strategy would be to integrate movement characteristics of single dots such as absolute dot velocity or size of the dot’s circular path, whereas the global strategy would be to detect coherently moving dots along the stimulus’ rotational axis. Pigeons are generally very good at differentiating moving from stationary stimuli [41] or in categorizing between velocities [42], but show rather poor performances in detecting coherent movements [28].

In a series of conditioning experiments, we tested six homing pigeons for their ability to detect the rotational centre of a rotating dot pattern. After it was clear that pigeons could learn the task, we specifically tested four strategies pigeons could use to solve it:

1. Do pigeons exploit the symmetry of dot patterns and detect the patterns' centre of mass rather than the centre of rotation?
2. Do pigeons search for slowly moving dots in the rotating dot pattern as a signal for the position of the rotational centre?
3. Do pigeons integrate between the rotational velocities of single dots in the rotating dot pattern, such that the presence of dots very close to the rotational centre should facilitate the detection of the pattern’s rotational centre?
4. Can pigeons avoid a local search strategy based on movement characteristics of single dots in the rotating dot pattern? In this case, their pecking response should not be influenced by the presence of stationary dots in the rotating dot pattern other than in the pattern’s rotational centre.

Thus, the first aim of the present study is to use an operant conditioning paradigm to test whether homing pigeons can learn to detect a centre of rotation in an arbitrary dot pattern. The second aim of this study is to investigate the perceptual strategies homing pigeons use to detect a rotational centre of a moving dot pattern.

## Methods

### Subjects

The experiments were carried out with six naïve adult homing pigeons (*Columba livia f*. *domestica*). Between the experiments, the birds were kept in individual wire mesh cages in a group room with visual contact to each other in a constant 12 h light dark cycle. The birds obtained water *ad libitum* and their weight was maintained at 85–90% of their free feeding weight. The experiments were approved by a national ethics committee of the state of North-Rhine-Westphalia, Germany (LANUV NRW, permit number AZ:8.87–50.10.37.09.277).

### Apparatus

The pigeons were trained individually in a custom-made operant conditioning chamber (Skinner-Box) with a size of 38 x 38 x 42 cm. The backside of the box was equipped with an active matrix TFT LCD touch display (Elo 1515L, Tyco Electronics, average luminance 70 cd/m², 1024 x 768 pixels, 75 Hz), so that the bird had access to an area of 23.6 x 20 cm of the screen. A feeder was positioned centrally beneath the screen. The LED house light, feeder illumination, stimulus protocol and data recording were controlled by functions of the Biopsychology Toolbox [43] in Matlab R2009a.

### Training

Initially, the birds were trained in an auto-shaping procedure to peck onto a stimulus shown on the screen to receive a food reward. The stimulus was a filled white circle of 1 cm in diameter in the centre of the black screen and was presented for 5 s or until a peck had occurred on the stimulus. When the pigeons reliably responded to the stimulus in at least 70% of the trials they were transferred to a continuous reinforcement schedule where one correct peck was sufficient for being rewarded (FR1) and the stimulus size was concurrently reduced to 0.35 cm in diameter. After the pigeons again showed a stable pecking response in at least 70% of the trials, this small stimulus became the start key on which the birds had to peck first within 20 s to initialise each trial. Initially, the same rotating stimulus was presented in each trial consisting of two bluish dots of 0.4 cm in diameter that rotated with a rotational speed of 7.2 degrees per second around a white dot (0.35 cm diameter) on a black background. The birds had to peck on the white dot within 20 s. In the course of two month, the number of dots in a pattern, the location of the pattern on the screen, the number of patterns per session and the rotational speed of the pattern were consecutively modified so that eventually, during one session, each trial presented one of four randomly selected rotating patterns consisting of varying numbers of small (0.2 cm diameter) bluish dots on a black background. In each trial, the rotating dot stimulus was presented for 20 s or until a peck into the rotational centre of the pattern had occurred. The patterns had an overall size of 10 x 10 cm and rotated around itself with a rotational velocity of 180 degrees per second at randomly chosen positions on the black screen. The rotational movement was achieved by updating a stack of 100 gif-frames that were rotated by 3.6 degrees counter-clockwise to the preceding frame every 0.02 s. We were aware of the fact that the resulting frame rate of 50 Hz was far below the critical flicker frequency (CFF) in pigeons, which can be up to 145 Hz under bright illumination [44]. However, during stimulus presentation, the LED house light was switched off and the light intensities emitted by the stimulus on the computer screen usually ranged between 2.8 ± 0.6 lux (mean ± sd) at a distance of 1 cm from the screen. Under such dim light conditions, the CFF of pigeons should be comparable to that in humans [45] so that a possible flickering of moving stimuli on a video screen should not cause disturbances to the birds [28,46].

In the rotational centre, the rewarded area (pecking field) of 1 x 1 cm in size was initially indicated by a small (0.2 cm diameter) white dot. By reducing the opacity level in steps of 50%, 25%, 10%, 7%, 5% and 3%, the white dot gradually faded away so that in the end of the training, the pigeons had to find the rotational centre of the dot pattern without any indications of the pecking field.

### Testing

Each test session lasted up to one hour or until 80 rewards were gained. During the test sessions, novel dot patterns that were not used during training were presented to the pigeons. In each trial, one of four different stimuli was randomly selected and presented at random locations set by the computer so that the rotational centre could be located anywhere on the touch screen. The stimulus covered either a small portion of the screen (10 x 10 cm) or the entire screen (40 x 40 cm, experiment 1). In the large stimulus, parts of the pattern temporarily disappeared during the rotation so that the pigeons could not derive the rotational centre from the stimulus size or from the centre of mass of the stimulus. During any given session, the stimulus size was kept constant. The pigeons ran one session daily and they were weighed on a scale before and after each session. For each session, the overall performance (percentage of correct trials), number of rewards, number of not initialised and of unsuccessful trials, the coordinates of each peck and of the presented stimulus on the screen, gif-frame number of each peck, and peck times were automatically recorded by the computer.

### Statistics

For analyses, the first peck occurring at least 200 ms after stimulus initiation was defined as the pecking response of the individual pigeon to the presented stimulus in the given trial. This was independent of whether the given trial was successful (rewarded) or not. Since pigeons working for food rewards peck with their beaks slightly open [47] and because the pigeons in our experiments usually pecked more than once on the start key, the 200 ms delay time was necessary to avoid scoring multiple pecks onto the start key as a response to the consecutively presented stimulus. Reaction times of pigeons in visual search tasks typically vary between 200–500 ms depending on the complexity of the visual display and reinforcement ratio [48–50]. We assumed a reaction time of 300 ms and therefore considered a given peck to be a response to the pattern the pigeon saw 300 ms before the peck.

**Fig 1:**
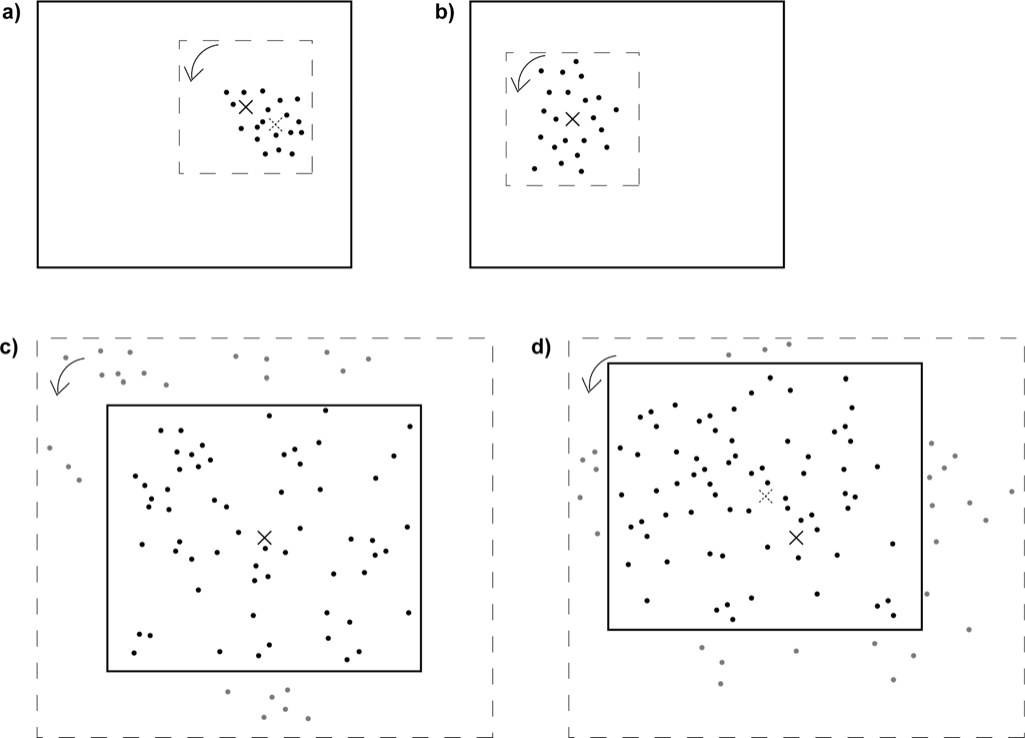
Relation between the centre of rotation (solid crosses) and centre of mass (dashed crosses) in small 10 x 10 cm dot patterns (a + b) and in large 40 x 40 cm dot patterns (c + d) covering the whole screen. Solid squares represent the limits of the computer touch screen; dashes squares indicate the stimulus boundaries, which were not visible to the pigeons because both the background of the screen as well as the background of the stimulus were presented in black. The centre of rotation was always located in the centre of the stimulus boundaries (dashed squares) but the stimulus could appear anywhere on the computer screen. In the large stimuli (c + d), the pattern exceeded the screen limits. Arrows indicate the overall rotation direction of the stimulus. The more asymmetric the dots were arranged in the rotating dot pattern with respect to its rotational centre (solid crosses), the greater the distance was between the rotational centre and the centre of mass (dashed crosses). In symmetric dot patterns, the rotational centre and the centre of mass coincided (b) whereas in the large stimuli (c + d), where dots were distributed all over the screen, the centre of mass of the visible part of the stimulus always coincided with the centre of the screen, no matter where the patterns' rotational centre was located.

The peck locations were analysed relative to the centre of the rotating dot pattern (rotational centre) and relative to the centre of the dotted stimulus area. This area centre corresponded to the centre of mass of the dot pattern. Thus, the location of the centre of mass always varied with the distribution of dots over the whole 10 x 10 cm stimulus area (Fig. 1). The more asymmetric the dots were distributed over the stimulus area relative to the rotational centre, the more separated the rotational centre and the centre of mass were from each other (Fig. 1a). Contrary, in symmetric patterns the rotational centre and the centre of mass coincided (Fig. 1b). For the large stimuli in experiment 1, the centre of mass was defined as the centre of the screen since the dot pattern stretched beyond the limits of the screen. Thus, as long as the dots were mostly evenly distributed over the screen (for the visible part of the dot pattern) the dot pattern's centre of mass corresponded to the screen centre which was also the location of the previously shown start key (Fig. 1c).

For each individual pigeon, the median distances of all first pecks recorded under the given condition were calculated relative to the rotational centre of the dot pattern, its centre of mass or the screen centre and compared by a Wilcoxon signed-rank test. Subsequently, the equivalent group mean distances with standard errors (SE) for each tested condition were calculated and compared by a paired t-test. Comparisons of more than two conditions were performed on the individual peck distances to the rotational centre by a Scheirer-Ray-Hare test (SRH-test) which was based on rank sums. If significant a *post hoc* multiple comparison test with a Bonferroni correction was computed. If needed, further statistics are described in the individual methods sections of the particular experiments.

## The Experiments

### Experiment 1: Are pigeons able to find the centre of a rotating dot pattern?

The first experiment tested whether pigeons were able to find the rotational centre of an arbitrary dot pattern. The general testing procedure is described above. The pigeons ran three test sessions containing six unfamiliar asymmetric patterns of the small stimulus size (10 x 10 cm) and five test sessions each containing four unfamiliar patterns of the large stimulus size (40 x 40 cm) covering the whole screen.

#### Results and Discussion

**Fig 2:**
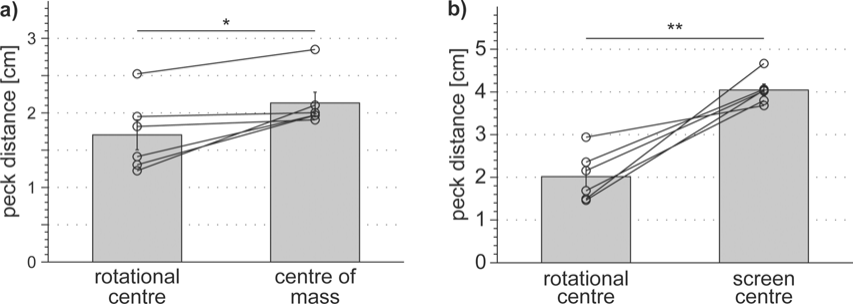
Pigeons significantly preferred pecking in the rotational centre of the rotating dot patterns over pecking in the centre of mass of both the 10 x 10 cm stimuli (a) and the 40 x 40 cm stimuli (b). Open circles depict individual median peck distances in cm for each pigeon; bars depict group mean peck distances in cm with standard errors. Paired t-test: * p < 0.05, ** p < 0.01.

The overall performance of the individual pigeons was 94 ± 5% correct trials relative to the total number of all initialised trials for the small stimuli and 63 ± 7% for the big stimuli. The individual median peck distances relative to the patterns’ centre of rotation were significantly smaller than the peck distances relative to the patterns’ centre of mass for the small stimuli (1.70 ± 0.20 cm vs. 2.13 ±0.15 cm; paired t-test: t = -3.19, sd = 0.33, df = 5, p = 0.02; Fig. 2a) and relative to the centre of the screen for the large stimuli (2.02 ± 0.24 cm vs. 4.04 ± 0.14 cm; paired t-test: t = -6.03, sd = 0.82, df = 5, p = 0.002; Fig. 2b). Individual peck distances to the rotational centre varied significantly between the different presented dot patterns for both the small (SRH-test: H(stimulus) = 21.57, df = 5, p < 0.001) and the large stimuli (SRH-test: H(stimulus) = 519.79, df = 19, p < 0.001). Individual pigeons also responded differently to the different presented patterns both for the small stimuli (SRH-test: H(pigeon) = 125.68, df = 5, p < 0.001 and H(pigeon*stimulus) = 59.93, df = 25, p < 0.001) and for the big stimuli (SRH-test: H(pigeon) = 187.24, df = 5, p < 0.001 and H(pigeon*stimulus) = 142.79, df = 95, p = 0.001).

The results of the first experiment indicate that the pigeons were able to find the rotational centre in unfamiliar dot patterns. Overall, their performances lay well above chance level. Our pigeons did neither simply peck somewhere on the computer screen nor did they choose to peck into the centre of mass of the rotating stimulus. The pigeons were even able to solve the task when parts of the pattern disappeared beyond the screen, although with lower performance levels. This drop in performance is possibly related to some extent to the fact that the rewarded pecking field of 1 cm² covered 1% of the stimulus area in the small, but only 0.21% in the large stimulus array. Thus, it was easier to find the pecking field in small stimulus configurations.

These results imply that our conditioning paradigm worked. However, we noticed a considerable scatter in the individual peck distances relative to the rotational centre depending on the presented dot pattern. In the next experiments, we therefore modified single aspects of the dot patterns to figure out *how* the pigeons managed to find the rotational centre.

### Experiment 2: Are pigeons using dot density as an indicator for the rotational centre?

**Fig 3:**
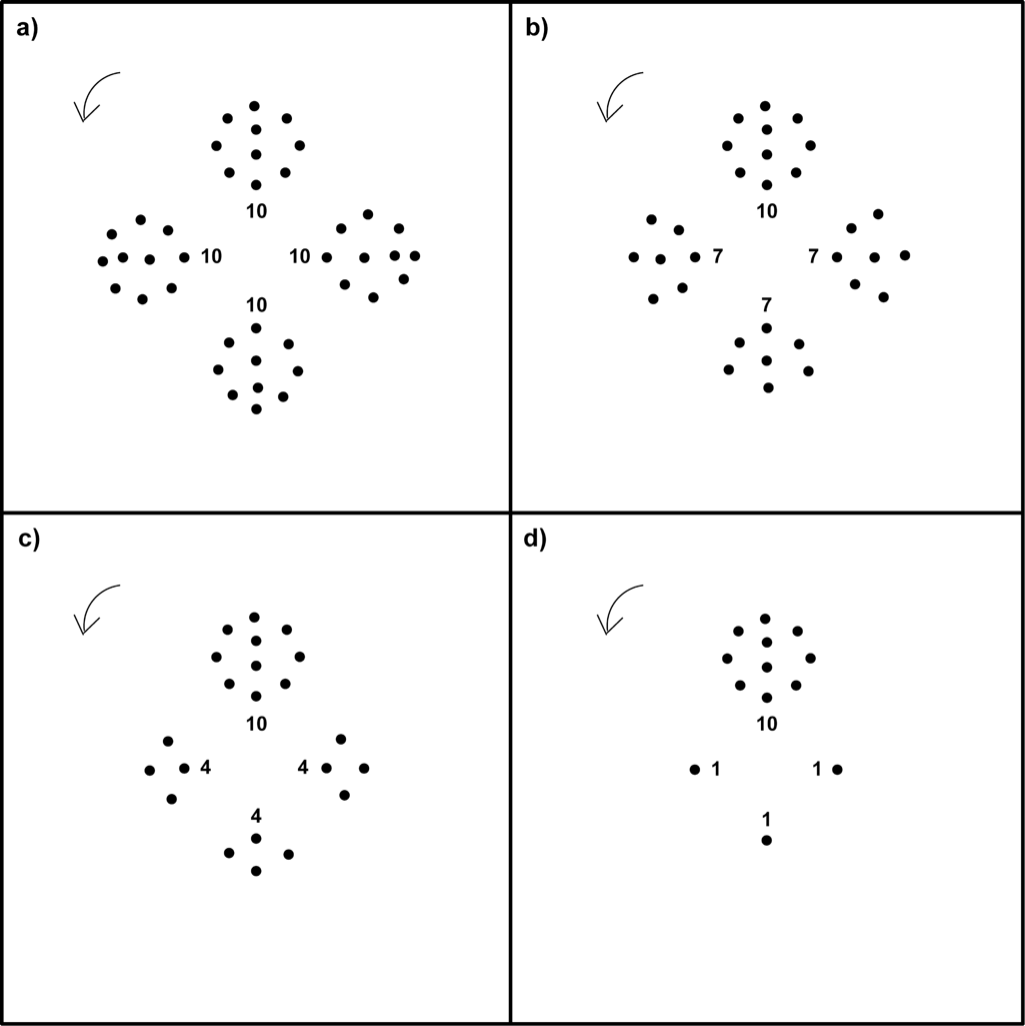
Example of a dot pattern that was part of a series of four symmetry conditions rotating counter clockwise (arrow). In the basic pattern (a), the four dot clusters containing ten dots each were symmetrically arranged in around the patterns' rotational centre. In the next three conditions (b-d), the upper dot cluster always maintained its ten dots whereas, in the remaining three dot clusters, the number of dots was reduced to seven dots each (b), four dots each (c) or one dot each (d). Note that the overall geometrical dot configuration of the remaining dot cluster remained identical in all conditions. The dot closest to the pattern's rotational centre of each dot cluster was always located 1.4 cm from the rotational centre. In the symmetrical condition (a), the pattern's centre of mass coincided with the pattern's rotational centre (0.09 ± 0.03 cm; mean ± sd; n = 4), but in the three asymmetrical conditions, the location of the pattern's centre of mass increasingly shifted towards the largest dot cluster with distances from the rotational centre of 0.32 ± 0.05 cm (b), 0.69 ± 0.10 cm (c) and 1.58 ± 0.23 cm (d). In contrast, the centre of the largest dot cluster kept its position relative to the pattern's rotational centre in all four symmetry conditions (2.17 ± 0.33 cm).

The first experiment revealed that the pigeons were able to locate the rotational centre of the rotating dot pattern but did not indicate their preferred perceptual strategy. One strategy could be to simply peck at the densest part of the pattern. To test this possibility, we varied dot densities in the second set of experiments. Fig. 3 shows an example pattern series. The basic pattern contained four clusters of ten dots each (Fig. 3a). The four clusters were arranged symmetrically around the patterns’ rotational centre (90° apart from each other). The dot closest to the rotational centre in each cluster was positioned 1.4 cm from the rotational centre. This distance was chosen to clearly separate between pecks into the rotational centre or within a dot cluster. There was no dot located in the rotational centre itself. In this symmetrical basic dot pattern, the centre of mass and the rotational centre coincide at any rotational angle of the pattern. In the next three conditions (Fig. 3b-d), the centre of mass of the dot pattern became more and more separated from the rotational centre. This was achieved by reducing the number of dots in all but one dot cluster to seven, four and one dot, respectively. The remaining dot cluster always maintained its ten dots. Thus, in the most asymmetric condition (Fig. 3d), the centre of mass of the dot pattern and its rotational centre are separated by 1.58 cm ± 0.23cm (mean ± sd, n = 4), and the centre of the largest cloud of dots was always separated from the rotational centre by 2.17 ± 0.33 cm. Four different basic patterns were designed. Each pigeon participated in eight test sessions. In a given test session, the four versions of a single basic pattern were presented in a randomised fashion.

To evaluate the direction of the pigeons’ pecks, peck orientation angles relative to the location of the biggest dot cluster at the time when the peck was recorded were calculated for each individual peck and averaged for each individual pigeon in each symmetry condition by vector addition. From these data, a group mean orientation angle was calculated and plotted for each symmetry condition and tested for significance by a Rayleigh test for circular data [51].

#### Results and Discussion

**Fig 4:**
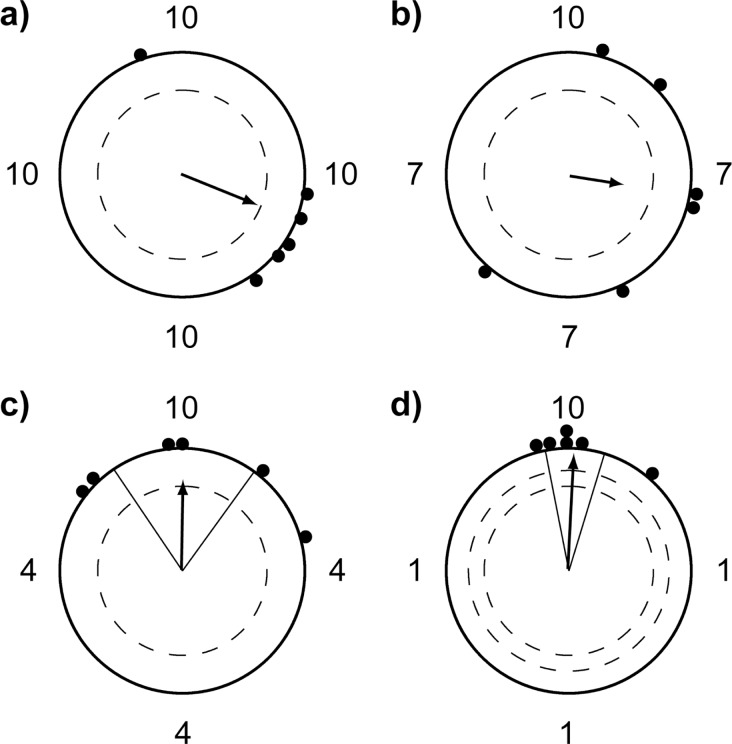
Peck orientations relative to the location of the largest dot cluster in the rotating dot pattern for each symmetry condition. In the first two conditions (a + b), the pigeons did not significantly bias their pecks towards a particular dot cluster (a: circular mean = 112°, r = 0.68, n = 6, p = 0.06; b: circular mean = 99°, r = 0.47, n = 6, p = 0.29). In the two more asymmetrical conditions (c + d), the pigeons significantly directed their pecks towards the largest cluster of dots (c: circular mean = 1° ± 38°, r = 0.74, n = 6, p = 0.03; d: circular mean = 3° ± 14°, r = 0.95, n = 6, p < 0.01). Numbers indicate the size of the dot clusters with the largest dot cluster always depicted at the top of each diagram (corresponds to 0°). Filled circles are the mean peck orientation angles relative to the largest dot cluster for each bird. Arrows indicate the group mean peck orientation angles. The inner dashed circles represent the length of the mean vector required for 5% significance and in d) 1% significance of the peck directions according to the Rayleigh test. Solid lines flanking the mean vector in c) and d) indicate the 95% confidence intervals for the mean direction.

When the four dot clusters were mostly symmetrically distributed around the patterns’ rotational centre, the pigeons’ pecks were not biased towards a particular dot cluster (10:10:10:10 dots: Rayleigh test: mean = 112°, r = 0.68, n = 6, p = 0.06; Fig. 4a; and 10:7:7:7 dots: Rayleigh test: mean = 99°, r = 0.47, n = 6, p = 0.29; Fig. 4b) which indicates that the pecks were randomly distributed around the dot patterns’ rotational centre. In the two most asymmetric dot configurations, the pigeons significantly biased their pecks towards the largest dot cluster (10:4:4:4 dots: Rayleigh test: mean = 1° ± 38°, r = 0.74, n = 6, p = 0.03; Fig. 4c; and 10:1:1:1 dots: Rayleigh test: mean = 3° ± 14°, r = 0.95, n = 6, p < 0.01; Fig. 4d).

**Fig 5:**
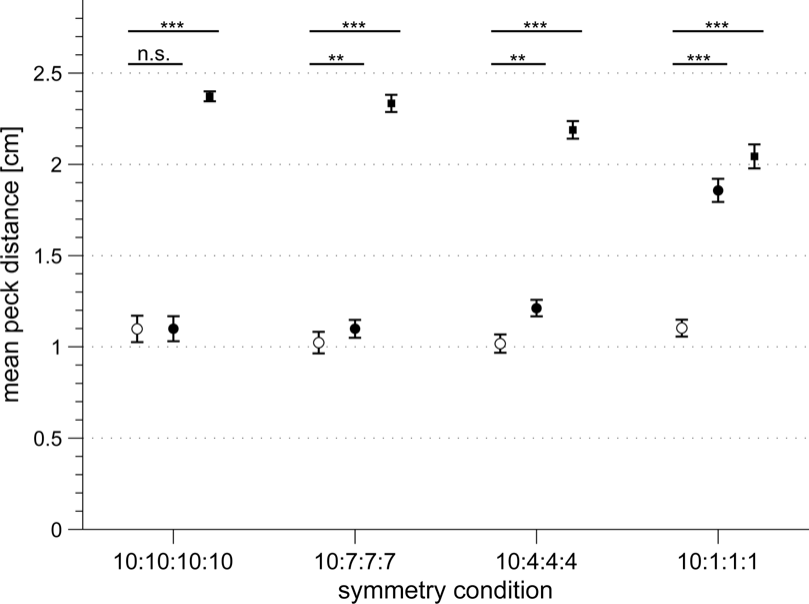
Pigeons significantly preferred to peck in the patterns' rotational centre (open circles) over pecking in the patterns' centre of mass (filled circles) and over pecking in the centre of the largest dot cluster (filled squares) in each symmetry condition. The peck distances are shown as group mean peck distances in cm with standard errors and are compared by a paired t-test in each condition (** p < 0.01, *** p < 0.001). In the symmetrical condition, the dot patterns' centre of mass coincided with the patterns' rotational centre but shifted towards the largest dot cluster with increasing asymmetry of the pattern. In contrast, the location of the centre of the largest dot cluster remained constantly positioned 2.17 ± 0.33 cm (mean ± sd) away from the patterns' rotational centre (see Fig. 3).

Surprisingly, the median peck distances relative to the centre of the rotating dot pattern were not increased by this bias since they did not correlate with pattern symmetry (r = 0.0062, p = 0.977; open circles in Fig. 5) and the pigeons still significantly preferred the rotational centre over the centre of mass of the dot pattern (filled circles in Fig. 5). Thus, the actual peck positions of the pigeons were attracted towards higher dot densities, but still remained close to the overall rotational centre. Consequently, the pigeons also did not peck into the largest cloud of dots (filled squares in Fig. 5).

These results reveal that the varying density of dots had no influence on the absolute peck distances of the pigeons relative to the rotational centre of the dot pattern. However, high dot densities strongly affected the orientation bias of the pecks. This might possibly be related to the tendency of pigeons to peck at contrast rich areas or the edges of a presented stimulus [47] rather than into the middle of an “uniform” stimulus area (example peck locations in figures one and two in [52] or figure seven in [53]).

This experiment also provides an experimental estimate of the reaction times of our pigeons for this specific task. As the stimuli rotated counter-clockwise, a clockwise or counter-clockwise bias of the peck orientations would indicate a delayed or advanced pecking response of the pigeons. However, in the most asymmetric condition, the peck orientations precisely matched the orientation of the largest dot cluster. This indicates that the assumed reaction time of 300 ms [48–50] fits very well to the behavioural responses of our pigeons.

Despite the clear bias towards the larger cloud of dots, the pigeons were still able to solve the task and their peck distances relative to the centre of rotation did not increase with increasing asymmetry of the pattern. Therefore, we conclude that pigeons do not necessarily use dot densities to locate the rotational centre of a rotating dot stimulus even when dots in the rotating dot pattern are extremely biased towards one dot cluster.

### Experiment 3: Does the rotational speed affect the performance of the pigeons?

Reducing the rotational speed of the dot pattern leads to smaller movement distances of single dots of the pattern, which should make it harder to detect the rotational centre. Therefore, experiment 3 was designed to test if the rotational speed of the dot patterns influenced the ability of pigeons to find the centre of rotation. Our pigeons ran four test sessions and we used a mix of the same four dot patterns in all sessions. We defined eight speed categories (table 1) and each of the four dot patterns was presented in two different speed categories (one fast and one slow) during one given session. To avoid pattern effects, the pattern—speed assignment was re-mixed in the next sessions, so that in the end of the four sessions each pattern had been presented in each speed category to all pigeons. The rotational speed of the patterns was modified by the number of gif-frames per image (100–800 frames) and by the time for updating the gif-frames (0.02–0.15 s) resulting in different frame rates at different speed categories as listed in table 1. Parameters were set to achieve a smooth rotation of the stimulus trading off frame loading time by the computer. Pecks to the rotational fix point were not rewarded before the stimulus had been presented for at least 1.5 s to avoid random pecking in the slow rotating stimuli. To test for a correlation between rotational speed and the individual median peck distances relative to the rotational centre, a non-linear curve fit was performed.

**Table 1:** The rotational speed of the rotating dot patterns was modified by varying the number of gif-frames for a full 360° rotation resulting in smaller angular distances in° between two successive frames as well as by the frame update time in s resulting in slower frame rates in Hz.

rotational speed [°/s]	number of frames	rotation per frame [°]	update time [s]	frame rate [Hz]
**180**	100	3.6	0.02	50
**90**	100	3.6	0.04	25
**60**	100	3.6	0.06	16.7
**45**	200	1.8	0.04	25
**22.5**	200	1.8	0.08	12.5
**11.25**	400	0.9	0.08	12.5
**5.625**	800	0.45	0.08	12.5
**3**	800	0.45	0.15	6.7

#### Results and Discussion

**Fig 6:**
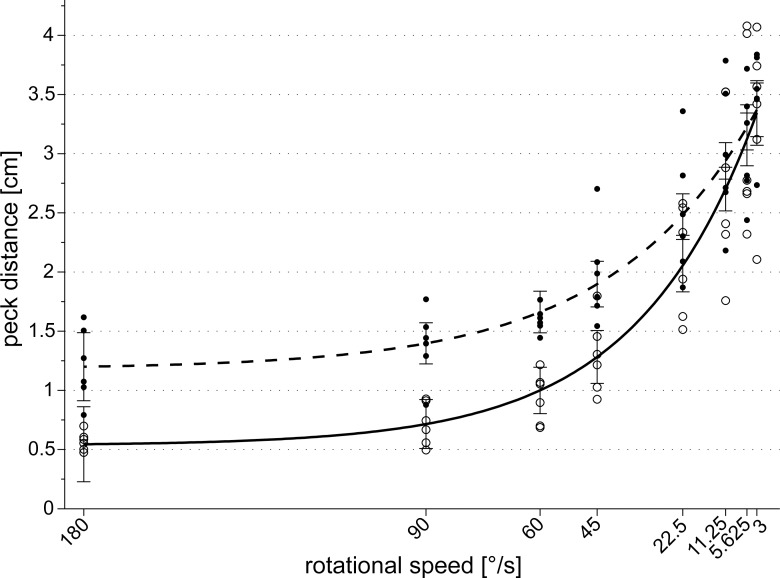
Decreasing the rotational speed of the dot pattern led to increased individual median peck distances relative to the pattern's rotational centre (open circles). The data points are best fitted by the exponential equation y = 0.53 + 3.09 * e^(-0.03x)^, r² = 0.84 (solid regression line). The individual median peck distances relative to the pattern's centre of mass (filled circles) also increased with decreasing rotational speed (dashed regression line: y = 1.18 + 2.37 * e^(-0.03x)^, r² = 0.81). Error bars represent the 95% confidence intervals for each predicted regression point.

The individual median peck distances to the rotational centre were significantly influenced by the rotational speed of the presented dot pattern (SRH-test: H(speed) = 910.23, df = 7, p < 0.001). The faster the pattern rotated, the closer the pecks were to the rotational centre (Fig. 6). Over all speed categories, individual pigeons showed different pecking responses (SRH-test: H(pigeon) = 38.25, df = 5, p < 0.001) but their peck distances relative to the rotational centre did not differ within a given speed category (SRH-test: H(pigeon * speed) = 40.11, df = 35, p = 0.254).

Fig. 6 depicts the pigeons’ performance as a function of the rotational speed of the presented dot pattern: The average peck distance to the rotational centre increased exponentially with decreasing rotation velocity following the equation y = 0.53 + 3.09 * e^(-0.03x)^; r² = 0.84.

At a speed of 5.625 degrees per second or less, there was no detectable difference between the pigeons’ group mean peck distance relative to the patterns’ rotational centre and relative to the patterns’ centre of mass (3.09 ± 0.31 cm vs. 3.07 ± 0.19 cm, paired t-test: t = 0.10, sd = 0.53, df = 5, p = 0.924; table 2). This implies that at this low level of rotational speed, the pigeons could no longer locate the rotational centre of the rotating dot pattern. Conversely, at speed levels above 5.625°/s, the pigeons significantly preferred the rotational centre over the centre of mass (table 2). However, since the frame rate (table 1) was 12.5 Hz or less in the conditions with a rotational speed of 22.5 degrees per second or less, we cannot exclude that the birds have no longer perceived the pattern as moving and that this could be the main reason, why they could no longer detect the centre of rotation.

**Table 2:** Decreasing the pattern's rotational speed resulted in increasing mean peck distances relative to the patterns' rotational centre.

rotational speed [°/s]	mean distance to centre of [cm]		paired t-test			
	rotation	Mass	t	sd	df	p
**180**	0.57	1.22	-5.92	0.27	5	0.0020**
**90**	0.72	1.39	-7.89	0.21	5	0.0005***
**60**	0.94	1.60	-7.99	0.20	5	0.0005***
**45**	1.29	1.97	-6.45	0.26	5	0.0013**
**22.5**	2.09	2.49	-2.94	0.33	5	0.0321*
**11.25**	2.74	2.98	-3.61	0.16	5	0.0154*
**5.625**	3.09	3.07	0.10	0.53	5	0.9238
**3**	3.34	3.48	-1.08	0.32	5	0.3306

At a rotational speed of 5.625 °/s and slower, the pigeons' mean peck distances relative to the rotational centre of the dot pattern were statistically indistinguishable from the pigeons' mean peck distances relative to the patterns' centre of mass.

* p < 0.05,

** p < 0.01,

*** p < 0.001

Our results are in line with previous studies examining the influence of rotational speed on the discrimination of rotation directions. Koban and Cook [54] also described an exponential loss of discrimination ability in pigeons with decreasing rotational speed. The performances of their pigeons were still above chance level even at their lowest rotational speed of 13°/s [54].

Another important issue concerning the methodology we used to create different rotational velocities should also be considered. We did not only increase the number of frames to achieve a full rotation resulting in a smaller angular difference between the dots of two successive frames, but also decreased the frame rate. Therefore, the rotation might have looked jerkier to the pigeons at lower rotational speeds because pigeons possess a higher critical flicker fusion frequency (CFF) than humans [44]. Thus, the task to find the rotational centre of the stimulus could have become more difficult not only because of slower rotational speeds but also because of possible flickering of the stimulus [45]. However, the CFF also decreases at lower light intensities [44] and keeping in mind that our stimuli emitted a very dim light intensity of about 2.8 ± 0.6 lux (see method section) and also that the spatial angle between two successive frames at low rotational velocities was about 0.45 degrees, we assume that flickering did not affect the performance of the birds.

### Experiment 4: Do pigeons search for slowly moving dots close to the rotational centre?

The previous experiments revealed that the rotational speed of the presented stimulus strongly affected the discrimination precision of the pigeons. As dots close to the rotational centre move with a slower relative speed pigeons could simply search for slow moving dots close to the centre of rotation. This would become more difficult with decreasing rotational velocities of the dot patterns. Furthermore, the pigeons were initially trained to peck at a stationary dot located in the rotational centre of the dot pattern. In the course of the training, that dot gradually faded away. Therefore, experiment 4 was designed to test if our pigeons simply pecked at slow moving dots located close to the patterns’ rotational centre.

**Fig 7:**
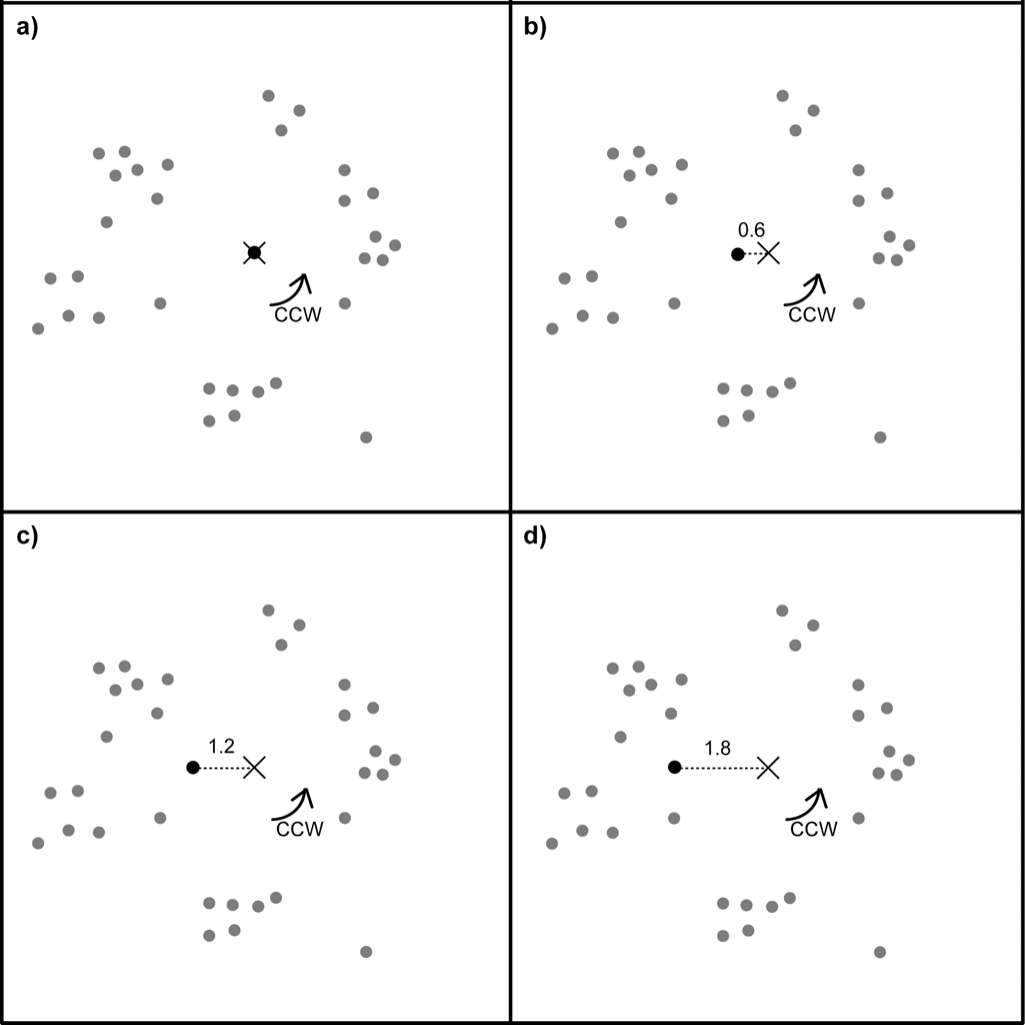
Example of a dot pattern series in which a single dot (depicted here in black) were located exactly in the pattern's rotational centre in the first experiment (a) and was then shifted relative to the pattern's rotational centre (indicated here by a cross) by 0.6 cm (b), 1.2 cm (c) or 1.8 cm (d) while the remaining dot configuration (depicted here in grey) remained identical. Other dots close to the pattern's rotational centre were located at least 1.6 cm away from the rotational centre.

We created five unfamiliar basic dot patterns for this experiment. The basic patterns were always the same except for the dot located closest to the rotational centre (the most central dot). The most central dot was either located exactly in the centre (in this case the dot did not move at all; Fig. 7a) or 0.6 cm, 1.2 cm or 1.8 cm away from the rotational centre (Fig. 7b-d), with all other dots being kept constant. Fig. 7 shows an example series of patterns. Each pigeon participated in five test sessions. In each of these single sessions, only one of the five pattern series was presented. Over the five sessions, each pigeon was presented with each of the five pattern series on one occasion.

#### Results and Discussion

**Fig 8:**
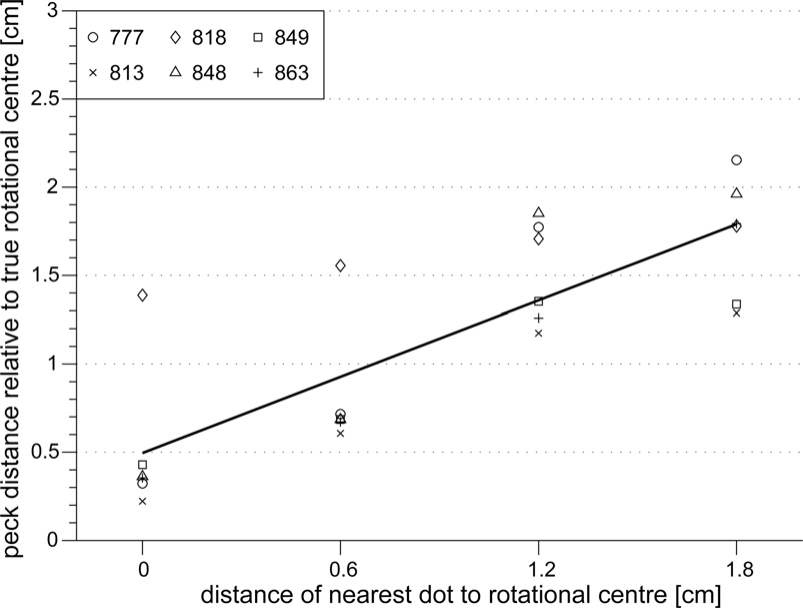
Peck distances of the pigeons relative to the centre of rotation increased (r = 0.81, p < 0.0001) when the distance of the dot located closest to the rotational centre increased. Individual median peck distances relative to the rotational centre in cm are depicted by individual symbols. Note that pigeon 818 (diamonds) showed generally greater peck distances to the rotational centre than all other pigeons (multiple comparison test: p < 0.001).

The individual peck distances relative to the rotational centre depended significantly on the location of the central dot (SRH-test: H(dot distance) = 798.12, df = 3, p < 0.001). However, there was individual variation in the peck distances and individual pigeons responded differently to the different pattern modes (SRH-test: H(pigeon) = 198.30, df = 5, p < 0.001 and SRH-test: H(pigeon * dot distance) = 128.05, df = 15, p < 0.001). Pigeon 818 showed generally greater peck distances relative to the rotational centre than all other pigeons (multiple comparison test: p < 0.001; diamonds in Fig. 8).

**Fig 9:**
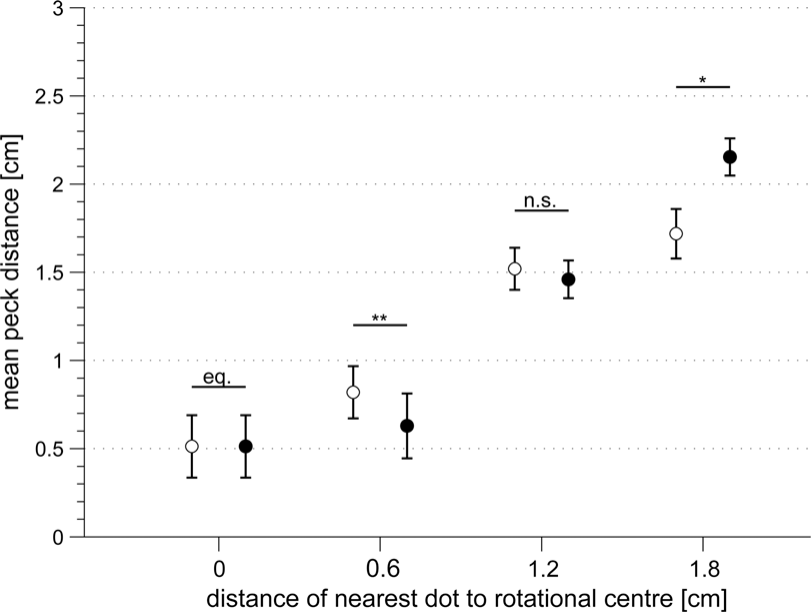
Dots close to the rotational centre of the dot pattern attracted the pigeons' pecks. Open circles depict the group mean peck distances relative to the rotational centre in cm. Filled circles depict the group mean peck distances in cm relative to the dot closest to the pattern's rotational centre. The peck distances were compared by a paired t-test for each dot distance category (* p < 0.05). Note that at a dot distance of 0 cm from the pattern's rotational centre, the peck distances are equal (eq.) by definition. Error bars indicate standard errors.

In addition, the individual median peck distances relative to the rotational centre increased linearly with increasing distance of the most central dot from the rotational centre (r = 0.81, p < 0.0001; Fig. 8). This indicates that the location of single dots near the rotational centre of the pattern might guide the pigeons’ pecks. With increasing drift of the most central dot away from the rotational centre of the pattern, the pigeons apparently compromised between the most central dot and the rotational centre. At a distance of 0.6 cm to the rotational centre of the pattern, the pigeons pecked closer to the most central dot than to the rotational centre (0.63 ± 0.18 cm vs. 0.82 ± 0.15 cm; paired t-test: t = 4.90, sd = 0.095, df = 5, p = 0.0045; Fig. 9). But when the most central dot was located at a distance of 1.2 cm to the rotational centre, the peck distances of the pigeons relative to the most central dot or relative to the centre of rotation did not differ (1.46 ± 0.11 vs. 1.52 ± 0.12 cm; paired t-test: t = -0.74, sd = 0.198, df = 5, p = 0.493; Fig. 9). In contrast, when the most central dot was located 1.8 cm from the rotational centre, the pigeons’ peck distance to the most central dot was significantly greater than to the rotational centre (2.15 ± 0.11 cm vs. 1.72 ± 0.14 cm; paired t-test: t = -3.10, sd = 0.344, df = 5, p = 0.027; Fig. 9), but the precision with which the pigeons detected the rotational centre decreased further.

These results indicate that dots located close to the patterns’ rotational centre influenced the pigeons’ pecking response. The dot closest to the centre of rotation moves less than dots located further away. At the rotational speed of 180°/s of our dot stimuli it should be very easy for the pigeons to identify slowly moving dots close to the patterns’ rotational centre by velocity categorisation [42]. In contrast, at a dot distance of 1.8 cm to the rotational centre of the dot pattern, the velocity differences between neighbouring dots are decreasing and it should become more difficult to find the rotational centre by velocity categorisation. However, at a distance of 1.8 cm to the rotational centre, also other dots of the pattern appear which can be similarly attractive to the pigeons. Thus, the greater peck distances relative to the most central dot in patterns with a minimum dot distance of 1.8 cm relative to the patterns rotational centre do not necessarily imply that, in this case, the pigeons actually pecked at the rotational centre or even changed their search strategy.

Our results also hint to another important aspect of the pigeons’ perceptual strategy. In dot patterns, the most central dot was located only 0.6 cm from the patterns’ rotational centre, the pigeons actually pecked at that particular dot instead of pecking at the rotational centre. This might have been augmented because the pigeons were initially rewarded for pecks at a stationary dot in the rotational centre of the dot pattern. Therefore, in the next experiments, we introduced additional stationary dots in the rotating dot patterns to investigate the pigeons’ search strategies.

### Experiment 5: Do pigeons peck at any stationary dot in a rotating dot pattern?

The previous experiments revealed that the pigeons’ pecking response might be strongly influenced by the presence of stationary or slow-moving dots close to the dot patterns’ centre of rotation. We therefore designed the next experiments to specifically investigate if the pigeons actually search for slowly or non-moving dots in the dot patterns rather than searching for the centre of rotation.

In the first test series, critical stimulus patterns that contained an additional non-moving dot inserted into the pattern at 1 cm, 2.5 cm or 4 cm from the rotational centre were mixed into normal test sessions as non-rewarded catch trials. Each pigeon participated in four sessions each including four catch trials. The catch trial stimuli were presented for 20 s or until five pecks had occurred somewhere on the stimulus area.

After this first test series, the birds were specifically trained to peck only at the rotational centre of the dot patterns and not at other fix points in the rotating dot pattern. The last training sessions consisted of patterns with no dot in the rotational centre but a varying number of alternative fix points occurring somewhere in the stimulus area. When the pigeons reached performances between 90% and 100%, a second test series was performed: eight of the same non-rewarded catch trials as in the first tests were randomly inserted into other training sessions and each pigeon ran four sessions with eight catch trials each.

Contrary to all other experiments in our study, in this experiment, all catch trial pecks were included in the individual analyses, resulting in 60 pecks per pigeon before training and 120 pecks after training.

#### Results and Discussion

**Fig 10:**
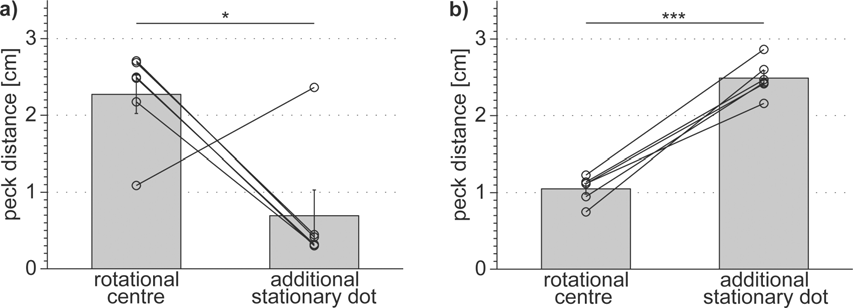
All but one pigeon significantly preferred to peck at a stationary dot that was additionally introduced into the rotating dot pattern (a). After specific training to ignore eccentric stationary dots, all of the pigeons significantly preferred pecking in the rotational centre over pecking at the additional stationary dot (b). Bars depict the group mean peck distances in cm with standard errors; open circles represent the individual median peck distances in cm. The group mean peck distances were compared by a paired t-test (* p < 0.05, *** p < 0.001).

During the first catch trial tests, the pigeons pecked significantly closer to the extra stationary dot than to the centre of rotation (0.69 ± 0.33 cm vs. 2.27 ± 0.25 cm; paired t-test: t = 2.75, sd = 1.41, df = 5, p = 0.040; Fig. 10a). The individual peck distances relative to the rotational centre depended significantly on the distance of the additional stationary dot from the rotational centre (SRH-test: H(fix point) = 138.55, df = 2, p < 0.001), but the individual pigeons responded differently (SRH-test: H(pigeon) = 51.72, df = 5, p < 0.001 and SRH-test: H(pigeon * fix point) = 51.28, df = 10, p < 0.001). Pigeon 818 pecked significantly closer to the rotational centre than all other pigeons (least significant difference test: p < 0.001, Fig. 10a), and it also pecked significantly closer to the rotational centre than to the centre of mass of the dot pattern (1.09 ± 0.85 cm vs. 1.53 ± 1.16 cm (median ± interquartile range), Wilcoxon test: signed rank = 213, z = -5.53, n = 64, p < 0.0001).

After specific training to ignore additional stationary dots in the rotating dot patterns, the pecking response of the pigeons reversed. All pigeons learned to significantly prefer the rotational centre over the additional stationary dot (1.05 ± 0.07 cm vs. 2.49 ± 0.10 cm; paired t-test: t = -12.46, sd = 0.28, df = 5, p < 0.001; Fig. 10b) and the rotational centre over the centre of mass of the dot pattern (1.05 ± 0.07 cm vs. 1.45 ± 0.09 cm; paired t-test: t = -10.41, sd = 0.10, df = 5, p < 0.001). The peck distances relative to the rotational centre still varied between the pigeons (SRH-test: H(pigeon) = 46.06, df = 5, p < 0.001) but did not depend on the distance of the extra stationary dot to the dot patterns’ rotational centre (SRH-test: H(fix point) = 4.29, df = 2, p = 0.117).

These results reveal that, in the first test series, most pigeons were attracted by the additional non-moving dot instead of by the rotational centre of the dot pattern. This implies a local search strategy based on detailed features of the rotating dot pattern. This might be a result of the learning history of the pigeons, which were initially trained to peck at a dot in the rotational centre of the pattern, i.e. a non-moving dot. However, after specific training to ignore stationary dots except if it was in the dot patterns’ rotational centre, all pigeons were able to detect the rotational fix point of the whole rotating pattern. They did not simply peck at stationary dots anymore. This might indicate a shift from a local perceptual strategy towards a more global strategy.

Previous studies have indicated that pigeons generally yield visual precedence for local cues [31,35], despite of their general ability to also use global, configurational cues [38–40]. We have shown that pigeons can be trained to change their search strategy and to ignore the local cues such as a stationary dot that stands out of the rotating dot pattern. This finding is comparable to a local-to-global strategy shift as found in other studies [39,40]. Troje and Aust [30] trained pigeons to discriminate a left-facing from a right-facing biological motion light-dot figure and demonstrated that most pigeons relied on motion characteristics of single dots (local cues) while a few of their birds adopted to a global analysis strategy. Thus, local vs. global perception not only depends on task properties and learning history but also includes an individual component [30].

From the results of our fifth experiment, however, we can unfortunately not determine the exact perceptual strategy our pigeons shifted to after the specific training. They could either be detecting the overall coherent dot movement [28] or they could be categorising between velocities [42], since both would be generally applicable for detecting a rotational centre.

## General Discussion

Our results reveal that homing pigeons can be trained to detect the rotational centre in a rotating dot pattern. They can do so by ignoring distractors like dot density, dots close to the centre, and eccentric, stationary dots. But all of these distractors affect the perceptual choices of the animal to some extent. Overall, most pigeons give visual precedence for local stimulus information such as the movement characteristics of single dots in the rotating dot pattern. This visual precedence for slowly or non-moving dots might have been favoured by the early training procedures. But after specific training, all pigeons were able to locate the centre of rotation even in the presence of other stationary dots.

It is interesting that one individual (pigeon 818) was never distracted by slowly moving or stationary dots at all. Pigeon 818 might have applied a global strategy from the start while being less precise as the other pigeons in indicating the rotational centre. More complex and thus time-consuming memory processing of global stimulus information as suggested by Cavoto and Cook [31] could have reduced the precision in indicating the rotational centre in this pigeon compared to the pigeons that just searched for a stationary dot.

Taken together, we can conclude from our experiments that pigeons can actually find the rotational centre of a rotating dot pattern and do not just peck at particular dots in the pattern. Both velocity categorisation by local stimulus cues as well as coherent movement detection based on global information would be principally appropriate strategies to detect a rotational centre.

In experiment 3, we observed a strong velocity dependence of our pigeons on their ability to locate the rotational centre. It is highly likely that the pigeons perceived our stimuli with their frontal visual field since we presented the dot patterns directly in front of them on a computer touch screen. The frontal visual field has a high visual acuity but a poor temporal resolution and has probably particularly evolved for pecking food at the ground [55]. This mechanism of visual processing with the frontal visual field supposedly favoured visual precedence for local stimulus information in our experiments [31] and might also be an explanation for the strong velocity dependence of the pigeons’ performances.

The ecological framework of our conditioning study originated from celestial compass orientation in migratory birds, and we propose that the centre of celestial rotation in nature should be detected mainly by the lateral visual field of birds which presumably has evolved for predator detection and flight control and is also more sensitive to motion detection [55–57]. Information from the lateral visual field is known to be processed within the thalamofugal visual pathway in pigeons [58] and zebra finches [59], and it is interesting that the thalamofugal visual pathway is already known to process magnetic compass information in European robins and garden warblers [4,60–63]. In some birds of prey, however, the Wulst is more concerned with the frontal field [64]. At present it is not known if lateral field representation and magnetic compass processing overlaps within the Wulst in night-migratory songbirds.

From the literature, it seems that the slowest velocities that visual neurons can still resolve range between 0.5 to 5 degrees of visual angle per second. This was measured in electrophysical recordings from the accessory optic system (AOS [65,66]; see [28] for summary). In contrast, the very slow celestial rotation is about 0.0042 degrees per second. This, together with the strong velocity dependence we observed in our experiments either implies the involvement of a different visual brain pathway than the AOS and/or makes it rather unlikely that birds perceive the celestial movement *per se*. For detecting the centre of celestial rotation, we propose a movement independent "snapshot strategy" instead. Since stars near the centre of celestial rotation move along smaller arcs than stars located further away, birds could, at one time, observe the star pattern relative to a stationary reference cue, which could be any landmark, and could compare it with the geometric constellation of the stars to the same reference at later times. Integrated over time, birds could then derive the centre of celestial rotation. Considering birds' apparent cognitive precedence for local information [31] and the importance of objects near the rotational centre revealed in our experiments, we suggest that stars or star patterns in the circumpolar region and/or the stationary Polar Star itself could be more important to young migratory birds, when they acquire their star compass, no matter how their exact perceptual strategy works. This idea is supported by the early behavioural experiments of Emlen [18].

The “snapshot strategy” would require the ability to recognise and to mentally rotate objects or patterns. Indeed, pigeons are able to compare rotated objects [67–70] and to recognise objects when they are presented in an unusual view [71]. Pigeons can even recognise rotated objects in a more efficient way than humans [72–74] and can also perform mental rotation [75]. Mental completion of geometric shapes [76] and objects [77] could facilitate star pattern comparison when parts of the pattern disappeared behind the horizon or clouds. Furthermore, Wallraff [78,79] showed in his classical conditioning experiments that mallards (*Anas platyrhynchos*) can recognise star patterns projected upon a planetarium sky independently from view, time of day and season. All these findings imply that birds should generally possess the cognitive prerequisites to perform the “snapshot strategy”.

To sum up, our experiments show that pigeons can learn the principle of finding the rotational centre of an arbitrary rotating dot pattern in an operant conditioning task. We further show that pigeons can be trained to abandon a local perceptual strategy they had initially been rewarded for and that they seem to be able to shift their attention to global rotational cues.

## Supporting Information

S1 Table Complete raw pecking data of all individual pigeons for each experiment. Listed are the individual peck distances in cm relative to the location of the centre of rotation and relative to the corrected location of the centre of mass of the given dot stimulus where it was located 300 ms before the analysed peck occurred (see method section). For experiment 1b which used large 40 x 40 cm stimuli, the individual peck distances in cm relative to the screen centre are listed. Note that although the physical dimensions of the screen were 30.4 x 22.8 cm, the pigeons only had access to an area of 23.6 x 20 cm (see method section) which was not exactly centred. Therefore, the location of the "screen centre" in experiment 1b was defined as the centre of the accessible part of the screen relative to the left/bottom edge of the screen which was defined as 0/0. Additionally, individual peck distances in cm relative to specific features of the given stimulus such as the largest dot cluster in experiment 2, the most central dot to the rotational centre in experiment 4 or the additional stationary dot in experiment 5 are listed. Furthermore, the coordinates in cm of individual pecks, the coordinates in cm of the centre of rotation and of the centre of mass, and the coordinates in cm of other analysed pattern characteristics relative to the left/bottom edge of the screen (0/0) are given. For experiment 2 also the peck orientation angles in degrees relative to where the largest dot cluster was located 300 ms before the peck had occurred are listed.
(XLSX)
